# Genetic analyses verify sexually mature escaped farmed Atlantic cod and farmed cod eggs in the natural environment

**DOI:** 10.1111/eva.13688

**Published:** 2024-04-17

**Authors:** Per Erik Jorde, Terje van der Meeren, María Quintela, Geir Dahle, Alejandro Mateos‐Rivera, Marit Aase, Birgitta Norberg, Pål Næverlid Sævik, Pål Arne Bjørn, Kevin Alan Glover

**Affiliations:** ^1^ Institute of Marine Research Flødevigen Norway; ^2^ Institute of Marine Research Bergen Norway; ^3^ The Directorate of Fisheries Trondheim Norway; ^4^ Institute of Marine Research Tromsø Norway

**Keywords:** aquaculture, assignment analyses, cod farming, farmed escapees, microsatellites

## Abstract

Elucidating the effects of domesticated organisms escaping into the natural environment represents a topic of importance in both evolutionary and conservation biology. However, when excluding the abundant data on salmonids, there is a lack of knowledge on this topic for marine fish aquaculture, which continues to expand globally. In order to bridge this empirical gap, we investigated a suspected escape of sexually mature domesticated Atlantic cod from a commercial marine fish farm in northern Norway. This involved genotyping samples of fish from cages on the farm, putatively identified escapees and wild cod captured in the region and samples of recently spawned eggs collected in the sea. Genetic analyses confirmed a farmed ancestry of the suspected escapees, and significantly, 27% of the sampled cod eggs. Furthermore, statistical analyses revealed a strong reduction in genetic variation in all samples of the farmed cod, including low effective population size and high degree of siblingship. These results thus document the escape of sexually mature adult cod and the release of fertilized domesticated cod eggs into the natural environment. Although it is possible that some of the mature escapees spawned post‐escape, the fact that only a single egg of potential hybrid farmed × wild origin was identified, together with the high number of mature cod in the farm, points to within cage spawning as the primary source of these eggs. This suggestion is supported by oceanic particle‐drift modelling, verifying that transport of eggs between the farm and the egg sampling locations was plausible. This study represents a rare documentation of interaction between domesticated and wild populations for a marine fish, pointing towards potential impacts on the local wild population.

## 1 INTRODUCTION

In parallel with the continued growth of the human population, ever‐greater demands are being placed on the world's resources, including those linked to food and especially protein production. Aquaculture is practised in all regions of the globe and is one of the fastest growing food production sectors, thus potentially filling the gap between food production and global demand (Bostock et al., 2010; Naylor et al., 2021). However, despite underlying optimism surrounding the future of aquaculture, this form of food production displays a wide range of environmental challenges (Cao et al., 2007; Edwards, 2015; Taranger et al., 2015), raising questions of sustainability.

In many geographic regions, finfish aquaculture is a relatively new activity and relies on domestication and selective breeding of recently captured wild organisms. Aquatic husbandry thus differs from its terrestrial counterpart by breeding on species that have extant wild relatives, often present in the immediate areas surrounding aquaculture facilities. Containment is a reoccurring problem linked to most forms of aquaculture, especially when it involves production in net pens (Jensen et al., 2010; Soto et al., 2023), thus posing the risk that domesticated fish escape pens and interbreed with wild conspecifics. Escape of aquaculture fish into the wild may lead to both genetic (reviewed by Glover et al., 2017) and ecological (Bradbury et al., 2020) impacts on native populations. While this topic has been extensively investigated in the context of domesticated Atlantic salmon (*Salmo salar*) farming and the long‐term documentation of escapees interbreeding in wild populations, many of the same concerns are also raised for a range of other marine fish species subjected to aquaculture (Alvanou et al., 2023; Bekkevold et al., 2006).

Atlantic cod (*Gadus morhua*) is a commercially important demersal fish distributed across most of the North Atlantic. The species has a long history of (over)exploitation, which has contributed to well‐documented stock collapses in parts of its natural distribution (Myers et al., 1997; Pershing et al., 2015). A combination of dwindling abundance coupled with an established consumer market has contributed to commercial interests in cultivating this species (Puvanendran et al., 2022). Although production has not yet taken off in the same manner as, for example, in Atlantic salmon, there are ongoing and renewed interests in establishing this as one of the main aquaculture species in several countries in the North Atlantic, including Norway (Puvanendran et al., 2022). Atlantic cod is now regarded as partly domesticated (Otterå et al., 2018; Puvanendran et al., 2022; Teletchea & Fontaine, 2014), which may both assist its potential expansion in production in the near future, but at the same time causes it to genetically diverge from wild cod, and thus leads to potential negative impacts if they escape and successfully interbreed with wild conspecifics.

Due in part to their tendency to display net‐biting behaviour (Damsgård et al., 2012; Hansen et al., 2008), Atlantic cod are effective at escaping from net pens (Jensen et al., 2010). In addition, farmed cod can also escape from net pens via within cage spawning that releases fertilized eggs into the surrounding water masses (Jørstad et al., 2008; Varne et al., 2015). However, although farmed cod escapees have been identified with genetic methods in the natural environment (Glover et al., 2011; Jørstad et al., 2014), and there have been attempts to look at the impacts of escapees and interbreeding with wild conspecifics (Varne et al., 2015), few data are currently available on the short term presence and long‐term genetic impact of Atlantic cod escapes in the wild. This situation is also the case for other marine species currently cultivated, and as a result, management authorities on a global scale need more data on the occurrence and potential impacts of escapees from non‐salmonids.

**FIGURE 1:**
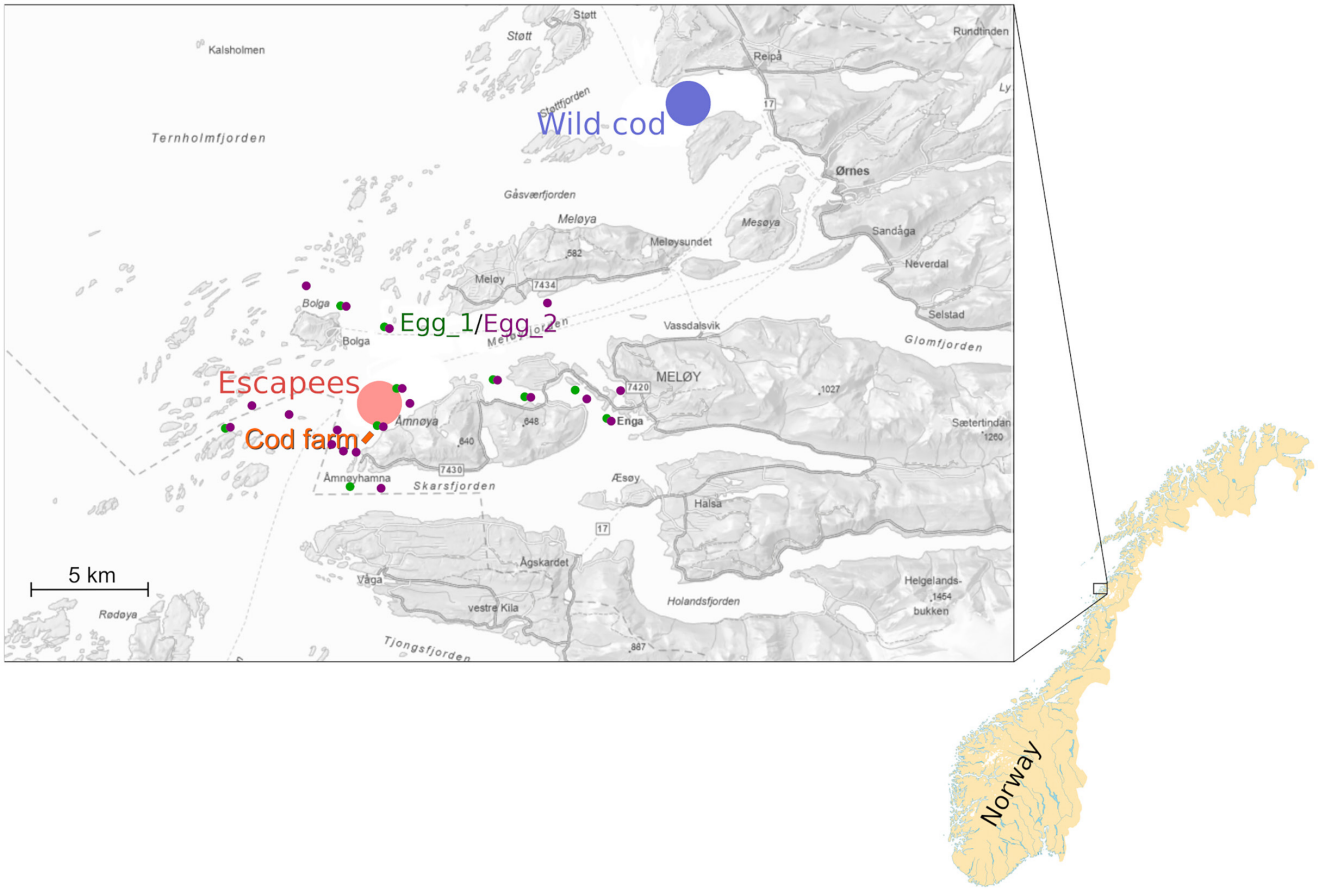
Location of the study area (insert) in northern Norway, depicting the location of the only cod farm in the region (orange), the area were suspected escaped farmed cod were collected for genotyping (light red), plankton net haul positions yielding cod eggs with sufficient DNA for genotyping (green and purple for the first and second egg survey, respectively), and the area were references samples of wild cod were collected (blue).

In January 2023, the Norwegian Directorate of Fisheries (NDF), responsible for fishery and aquaculture management and legislation in Norway, received reports of large numbers (initially 650, later adjusted up considerably) of adult cod of suspected farmed origin being harvested in marine fisheries located close to the Åmnøya island in Meløy municipality, northern Norway (Figure 1). According to the NDF, only one operational cod farm (Frosvika) was located in the region where the escapees were observed. Moreover, upon inspection of this farm, a high proportion of sexually mature cod was observed within the cages (NDF, 2023). As the Institute of Marine Research (IMR) routinely conducts the genotyping and analytical identification of aquaculture escapees in Norway (Glover, 2010), the situation in Meløy was followed up as a case study on the potential for the genetic impact of farmed cod on the natural environment.

The primary questions addressed in this study were to determine: (1) whether the putative escapees originated from the suspected nearby farm; (2) whether eggs of farmed cod could be identified within the water masses in the region and; (3) whether cod escapees had partaken in spawning with wild cod and producing farmed × wild hybrid offspring. These questions were addressed through genetic analyses of sampled farmed cod from cages, putative escapees captured by fishers, sampled wild adult cod and planktonic cod eggs collected in the region.

## 2 MATERIALS AND METHODS

### 2.1 Sampling of adult cod

Samples of adult cod captured in the vicinity of Frosvika cod farm in Meløy were collected by local commercial fishers using gill nets (Figure 1). As part of this commercial catch, suspected cod escapees were identified by the fishers due to their deviant body form and thus reported to the NDF, which gave permission to deliver such cod outside the commercial quotas. Over a few weeks, from 11 January to 31 March 2023, more than 14 tons of suspected escaped cod, or about 4000 individuals, were delivered to the local fish landing facility. From a subset of these, tissue samples, otoliths and other information were collected on site by the NDF and delivered to the IMR for analysis. Gill tissue was stored in 96% ethanol and used for genetic analyses (below), while otoliths were preserved in individual plastic bags prior to the assessment of its wild or farmed origin based on its shape. This assessment of origin was conducted by two independent and experienced otolith readers at IMR. Samples of wild adult cod were obtained from commercial fishers from the same general area for use as a ‘wild’ reference. Samples of farmed cod from the Frosvika farm itself were collected by the NDF from three cages (Table 1). All of the cod reared on the farm were reported to originate from the same broodstock (at Havlandet Marine Yngel AS).

**TABLE 1:** Cod samples analysed in the present study.

Sample name	Sample size	Genetic classification			
		Farmed	Wild	Potential hybrid	% farmed
Reference samples					
Farmed, cage 1	46	45	1	0	97.8
Farmed, cage 2	46	46	0	0	100.0
Farmed, cage 4	48	48	0	0	100.0
Wild adults, sample 1	69	0	69	0	0.0
Wild adults, sample 2	76	0	76	0	0.0
Target samples					
Eggs, sample 1	38	30	7	1	81.1
Eggs, sample 2	129	16	113	0	12.4
Suspected escapees	71	71	0	0	100.0
Sum	523	256	266	1	

*Note*: Samples are grouped into reference samples of farmed (3 cages) respective wild fish (2 samples), and target samples of eggs (2 surveys) and putatively escaped fish (1 sample) collected in the wild. Genetic classification of samples refers to consensus outcome of STRUCTURE and GeneClass. For a single egg a consensus between methods was not reached (intermediate STRUCTURE *q*‐value), assumed to be a farmed × wild hybrid and not used in the statistical analyses. One fish from the farm was apparently of wild origin.

### 2.2 Egg sampling

Two egg surveys, from 28 February to 2 March and 28 March to 30 March 2023, were carried out on 50 selected stations in the vicinity of the Frosvika cod farm and at known spawning areas for coastal cod east and west of the farm (Figure 1; Figures S1 and S2). The egg surveys were carried out during daytime over 3 days, using a WP2 plankton net with a 56 cm opening diameter, 500 μm mesh size and a cod end cup with 350 μm mesh window. The WP2 net was lowered to 50 m depth (or to the bottom in shallower water) and hauled vertically to the surface at about 0.5 m/s. Due to ice coverage during the second survey, two stations had to be omitted and one of them was replaced by a new station as close as possible to the omitted one. After net sampling and gentle sieving through 2000 and 750 μm to remove large and small zooplankton, eggs and remaining plankton were kept in 1‐L plastic bottles in a refrigerator at 4°C. All eggs were manually separated from the plankton in each sample using an Olympus SZ61 stereo microscope with a SZX2‐ILLT‐1‐5 transmitted LED illuminator stand light base and thereafter photographed at 8× magnification both in dark field (DF) and bright‐field (oblique) illumination modes to obtain enhanced contrast, colours and cell details of the eggs. After photographing, pure ethanol was added to the eggs to preserve them for DNA analysis. A total of 1900 eggs were collected in the first survey and 4772 in the second, of which 200 and 1128, respectively, were chosen for genetic analyses.

### 2.3 DNA isolation and genotyping

DNA from eggs was isolated as described by Mateos‐Rivera et al. (2020), while cod tissue was suspended in lysis buffer from the DNAdvance kit (Beckman Coulter, CA, USA) and processed using a Biomek i5 Automated Workstation (Beckman Coulter), following the manufacturer's instructions.

All samples (eggs and tissues) were genotyped with 21 microsatellite loci. Microsatellites were chosen as the marker of choice as extensive experience in genetic identification of farmed salmonids and gadoids (Glover et al., 2008, 2010, 2011) has illustrated that they are effective at detecting founder effects and drift that are rife in aquaculture and that they effectively resolve parent‐offspring and sibship relations (Duval et al., 2021; Quintela et al., 2016). Detailed genotyping conditions are summarized in Table S1. PCR products were diluted 1:20 and analysed on an ABI3730 sequencer (Applied Biosystems, MA, USA). Microsatellite alleles were scored using GeneMapper v6.0 (Applied Biosystems).

### 2.4 Genetic analyses

The starting step of the analytical process was to determine the farmed or wild origin of the target sample individuals, namely putative escapees and egg samples from the region. Thus, the individuals collected on the farm were regarded as the ‘farmed reference’ samples, whereas the adult wild fish collected in the sea (verified by otolith shape) were considered as the ‘wild reference’ samples. In contrast, escapees and eggs were regarded as ‘target samples’ and their direct assignment to their potential farmed or wild source on the basis of microsatellite genotypes was conducted using the Rannala and Mountain (1997) method, implemented in the program GeneClass 2.0 (Piry et al., 2004). In a separate step, Bayesian clustering implemented in STRUCTURE v.2.3.4 (Pritchard et al., 2000) was used under a model assuming admixture and correlated allele frequencies without using population information (LOCPRIORS) for the total set of 523 individuals, both reference and target samples. Ten runs of STRUCTURE were conducted at *K* = 2 (e.g. wild vs. farmed) with a burn‐in period consisting of 100,000 replications and a run length of 1,000,000 MCMC iterations. STRUCTURE was further used to compute posterior credible intervals (CI) for the ancestry of each individual. Runs were averaged with CLUMPP v.1.1.1 (Jakobsson & Rosenberg, 2007) using the FullSearch algorithm and the G' pairwise matrix similarity statistic and graphically displayed using barplots. A threshold of ancestry to cluster of *q* > 0.8 was used to classify individuals as belonging to that specific cluster. The consensus outcome of STRUCTURE and GeneClass allowed to separate sampled eggs and fish into farm‐ and wild origin samples for subsequent analyses.

Once samples were classified, the total number of alleles and allelic richness per sample were calculated using MSA 4.05 (Dieringer & Schlötterer, 2003). The observed (*H*_o_) and unbiased expected heterozygosity (*uH*_e_) as well as the inbreeding coefficient (*F*_IS_) were computed for each sample with GenAlEx v6.1 (Peakall & Smouse, 2006). Genotype frequencies were compared with Hardy–Weinberg expectations (HWE) and direction of deviance (i.e. heterozygote deficit or excess) was determined using the program GENEPOP 4.0.6 (Rousset, 2008), which was also used to calculate linkage disequilibrium (LD) within pairs of loci. Estimates of effective population size (*N*_e_) were obtained with the LD method implemented in LDNE (Waples & Do, 2008), using 0.01 as the lowest allele frequency. Confidence intervals (95%) around the point estimates were obtained by jackknife over loci. The false discovery rate (FDR) correction of Benjamini and Hochberg (1995) was applied to control for Type I errors when appropriate during multiple testings.

Genetic structure was assessed using pairwise *F*_ST_ (Weir & Cockerham, 1984), computed with Arlequin v.3.5.1.2 (Excoffier et al., 2005). Pairwise *F*_ST_ was visualized using a heatmap. The relationship among samples was also examined using the Discriminant Analysis of Principal Components (DAPC) (Jombart et al., 2010) implemented in the R (R Core Team, 2023) package *adegenet* (Jombart, 2008). Groups were defined by combining information of geographically explicit locations with the previously assessed farmed/wild ancestry of the individuals. To avoid overfitting, the cross‐validation function was used to determine the optimal of both number of principal components and discriminant functions to retain (Jombart & Collins, 2015; Miller et al., 2020).

Finally, family relationships between previously determined farmed individuals (eggs or adult fish) were investigated using COLONY v.2.0.5.1 (Jones & Wang, 2010), which implements full‐pedigree likelihood methods to simultaneously infer siblingship and parentage among individuals using multilocus genotype data. Analyses were run with no information on parental genotypes, assuming both male and female polygamy as well as possible inbreeding. The full‐likelihood model was chosen with run length and precision set to long and very high, respectively.

### 2.5 Oceanographic modelling

The oceanic current patterns were modelled to evaluate if pelagic eggs of farmed origin (cf. Figures S1 and S2), could have been spawned in the farm cages and dispersed by ocean currents to the locations where they were sampled, or alternatively, originated from escaped fish spawning in the wild. The hydrodynamic model is based on NorKyst800 (Albretsen et al., 2011), which uses the Regional Oceanic Modelling System (ROMS) (Shchepetkin & McWilliams, 2005) as its main engine. We used a refined version of the original NorKyst800 setup, with a horizontal resolution of 160 × 160 m and 35 terrain‐following vertical levels in a 312 × 145 km subdomain. The model uses historic atmospheric and river runoff data to recreate realistic currents and density profiles and has been validated against in situ measurements both within fjords and along the Norwegian coast (Asplin et al., 2020; Dalsøren et al., 2020).

Egg dispersion was simulated with the particle tracking model Ladim (Ådlandsvik & Sundby, 1994), using the Python implementation at https://github.com/bjornaa/ladim. Particles were allowed to drift at fixed depths of 5, 10 and 15 m only, as dynamic wind‐ and density‐driven mixing (Röhrs et al., 2014) would require a more thorough model validation, which was outside the scope of the present study.

## 3 RESULTS

### 3.1 Summary of reference and egg samples data

Of the 1328 eggs subjected to microsatellite screening, a total of 167 eggs, 38 from the first survey and 129 from the second, successfully amplified microsatellite DNA profiles. Eggs not amplified by the microsatellites were regarded as non‐cod eggs, and/or lacking sufficient DNA for identification, and disregarded in all following analyses.

In addition to the eggs, a total of 552 adult cod, including wild and farmed cod, were genotyped. Of these, 196 exceeded the threshold of missing data of 25% and were discarded from further analysis. Thus, the final data set for statistical analysis consisted of 356 adult cod and 167 cod eggs (Table 1). One locus, GmoC127, was dismissed due to poor amplification across samples and all statistical analyses were, therefore, based on 20 microsatellites (Table S1). The 523 individuals were segregated for a total of 443 alleles, ranging from 7 (at locus GmoC305) to 49 (locus GmoG25a).

### 3.2 Reference samples from the farm and wild

**FIGURE 2:**
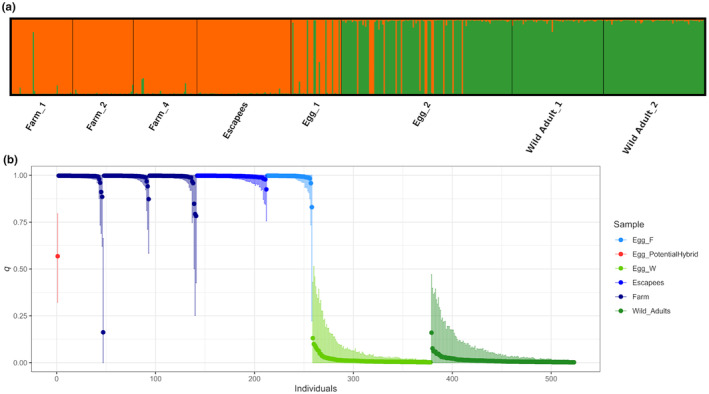
(a) Barplot representing the estimated proportion of individuals' ancestry to *K* = 2 clusters with STRUCTURE. Orange and green colours represent farmed and wild ancestry, respectively. (b) Individual ancestry to the farmed cluster (*q*) with 90% posterior credible intervals (CI: vertical bars). Eggs and adult individuals of farmed origin are depicted in blue colours, whereas green depicts wild origin. The individual egg marked in red is interpreted as a potential farmed × wild hybrid.

Prior to the identification of the putatively identified escapees and the cod eggs, the 20 microsatellites were tested for their ability to accurately identify ‘unknown’ samples as being of either farmed or wild origin. This ‘self‐assignment’ using the reference samples from the three farm samples and the two wild cod samples was conducted in GeneClass. This procedure correctly assigned all farmed individuals, except one, back to farmed reference samples and all wild fish to either of the wild reference samples, thus demonstrating an almost 100% correct self‐identification accuracy (Table 1). The single exception refers to one individual from Farm_1 (individual no. M1_18), which is assigned to wild cod with GeneClass. STRUCTURE verified these findings, yielding high probabilities (*q* > 0.80) for all reference individuals to their respective population of origin (Figure 2), with the exception of the M1_18 individual (blue dot at lower left in Figure 2b), which showed high probability (0.84) to wild ancestry. Credible intervals (CI: vertical bars in Figure 2b) were in general narrow with only a handful (mostly wild) fish overlapping 0.5. Wide CIs were not related to any obvious technical cause, such as the proportion of missing values (not shown).

### 3.3 Genetic identification of the target samples (putatively identified farmed escapees and cod eggs)

**FIGURE 3:**
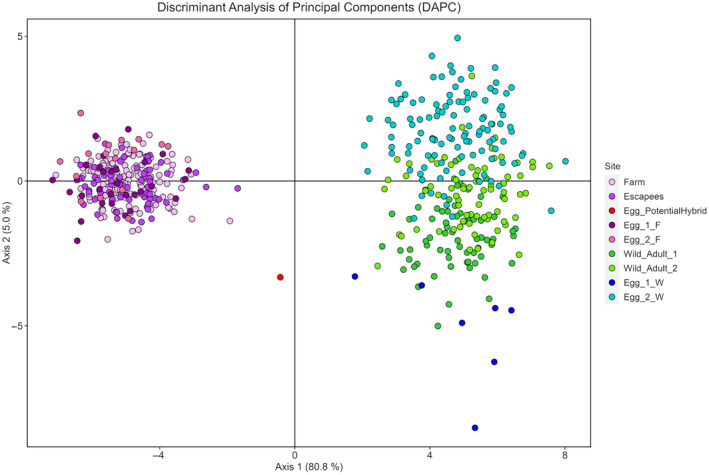
Genetic differentiation among cod samples assessed using Discriminant Analysis of Principal Components (DAPC) after retaining 200 principal components and three discriminant functions. Samples are coloured according to genetic assignments as in the legend.

GeneClass determined that all the 71 suspected escapees were indeed of farmed origin (67 of them with a score rank of 100%, three with >99.3% and one with 97.2%). In the first egg survey, 30 out of the 38 cod eggs were assigned to the farmed reference samples, while 8 assigned to the wild reference samples. In the second egg survey, 16 and 113 cod eggs were assigned to the farmed and wild reference samples respectively. The STRUCTURE barplot at *K* = 2 further confirmed that escapees displayed an unequivocal farmed profile, as did a number of eggs from both surveys 1 and 2 (Figure 3). Thus, 46 of the total 167 genotyped cod eggs (27.5%) showed farmed ancestry ranging between *q* = 0.833 and 0.998, whereas one egg displayed an approximately even (0.57/0.43) wild/farmed profile in STRUCTURE (red dot in Figure 2b) and was, therefore, regarded as a potential farmed × wild hybrid. For the remaining 120 eggs, a wild origin was inferred with estimated ancestry ranging between *q* = 0.869 and 0.998.

### 3.4 Genetic parameters observed within and among sample types

The above results yielded a consensus between the GeneClass and STRUCTURE methods classification of all target samples (Table 1), with the exception of the egg of apparent hybrid ancestry which was dismissed from statistical analyses. The following analyses were carried out after splitting the target samples into wild and farmed origins based on this consensus. In consequence, the 46 eggs of farmed origin (F) were distributed into samples Egg_1_F and Egg_2_F, with 30 and 16 individuals, respectively, whereas the 120 eggs of wild origin (W) were distributed into samples Egg_1_W and Egg_2_W, with 7 and 113 individuals each.

Genetic diversity greatly differed according to inferred ancestry, with samples of farmed origin scoring substantially lower on all measures of genetic diversity relative to samples of wild ancestry (Table 2). For example, allelic richness normalized to a sample size of *n* = 30 ranged between 7.0 to 7.3 for farmed and between 12.6 and 13.2 for wild cod, a reduction of 45% in the farmed stock. Likewise, average expected heterozygosity (u*H*_e_) ranged between 0.677 and 0.686 for farmed and between 0.772 and 0.792 for wild cod samples, a reduction of 13%. Samples of farmed and wild cod also differed in levels of linkage disequilibrium (LD) and yield substantially different estimates of effective population size based on those levels, from a few tens for farmed cod to several thousands for wild cod (Table 2).

**TABLE 2:** Summary statistics of genotyped samples.

Origin	Sample	*n*	No alleles	AR (*n* = 16)	AR (*n* = 30)	*H*_o_	u*H*_e_	*F*_IS_	Dev HW (FDR)	Dev LD (FDR)	*N*_e_ (95% CI)
Farmed	Farm_1	46	155	6.2	7.2	0.672 ± 0.043	0.677 ± 0.041	0.003 ± 0.015	5 (4)	23 (5)	63.8 (47.6–92)
Farmed	Farm_2	46	149	6.2	7.0	0.676 ± 0.049	0.680 ± 0.046	−0.005 ± 0.019	3 (3)	28 (4)	68.1 (53.7–90.5)
Farmed	Farm_4	48	150	6.3	7.0	0.654 ± 0.047	0.681 ± 0.042	0.049 ± 0.028	7 (2)	37 (12)	37.5 (30.6–47.1)
Farmed	Escapees	71	157	6.3	7.1	0.693 ± 0.044	0.682 ± 0.042	−0.028 ± 0.012	3 (1)	60 (16)	46 (37.6–57.6)
Farmed	Egg_1_F	30	142	6.3	7.3	0.690 ± 0.051	0.686 ± 0.043	0.001 ± 0.035	1 (1)	9 (0)	571.2 (141.5–∞)
Farmed	Egg_2_F	16	122	6.2	na	0.704 ± 0.047	0.711 ± 0.041	−0.032 ± 0.035	4 (2)	7 (1)	∞ (∞–∞)
Wild	Wild Adult_1	69	318	10.1	12.6	0.768 ± 0.043	0.783 ± 0.044	0.006 ± 0.015	5 (3)	9 (3)	∞ (2326.6–∞)
Wild	Wild Adult_2	76	327	10.2	12.9	0.724 ± 0.048	0.772 ± 0.049	0.061 ± 0.011	7 (6)	7 (2)	7877.7 (1048.9–∞)
Wild	Egg_1_W	7	128	na	na	0.750 ± 0.060	0.770 ± 0.045	−0.025 ± 0.048	0 (0)	0 (0)	∞ (∞–∞)
Wild	Egg_2_W	113	364	10.5	13.2	0.731 ± 0.039	0.792 ± 0.042	0.070 ± 0.012	9 (6)	13 (6)	4801.9 (1245.9–∞)

*Note*: Columns provide information on the origin of each sample (farmed or wild); sample name; number of individuals (*n*); total number of alleles (sum over 20 loci); allelic richness, AR, based upon 16 and 30 individuals; observed heterozygosity, *H*_o_ (mean ± SE); unbiased expected heterozygosity, u*H*_e_ (mean ± SE); inbreeding coefficient, *F*_IS_ (mean ± SE); number of significant deviations (in either direction) from HW and from linkage equilibrium (LD) at α = 0.05, both before and after (in parenthesis) False Discovery Rate (FDR) correction; estimated effective population size (*N*_e_) with 95% confidence interval calculated with jackknife over loci (negative numbers interpreted as infinite, ∞).

The pairwise *F*_ST_ tests revealed no or very low differentiation among samples of the same ancestry: 0 to 0.010 both for wild and farmed ones. *F*_ST_ in farm versus wild comparisons, on the contrary, ranged between 0.053 and 0.086, all significantly different from zero also after FDR correction (Table 3). The first axis of the DAPC (Figure 3), accounting for 80.8% of the variation, neatly discriminated the individuals according to their origin. Thus, fish and eggs of farmed ancestry occupy the left side of the figure while fish and eggs of wild provenance are on the right. The putative hybrid (red dot) occupies an intermediate position between both groups. The second axis (5%) slightly discriminated between eggs and adults of wild origin.

**TABLE 3:** Genetic differentiation between groups after redistributing sampled individuals according to their inferred farmed and wild ancestry.

		Farmed origin						Wild origin			
		Farm_1	Farm_2	Farm_4	Escapees	Egg_1_F	Egg_2_F	Adult_1	Adult_2	Egg_1_W	Egg_2_W
		*N* = 46	*N* = 46	*N* = 48	*N* = 71	*N* = 30	*N* = 16	*N* = 69	*N* = 76	*N* = 7	*N* = 113
Farmed	Farm_1	–	0.595	**0.026**	0.064	0.218	0.549	**0.000**	**0.000**	**0.000**	**0.000**
	Farm_2	0.000	–	0.034	**0.012**	0.624	0.319	**0.000**	**0.000**	**0.000**	**0.000**
	Farm_4	0.005	0.005	–	**0.031**	**0.019**	0.081	**0.000**	**0.000**	**0.000**	**0.000**
	Escapees	0.003	0.005	0.004	–	**0.001**	0.141	**0.000**	**0.000**	**0.000**	**0.000**
	Egg_1_F	0.002	0.000	0.007	0.010	–	0.329	**0.000**	**0.000**	**0.000**	**0.000**
	Egg_2_F	0.000	0.002	0.007	0.004	0.001	–	**0.000**	**0.000**	**0.000**	**0.000**
Wild	Adult_1	0.057	0.055	0.055	0.053	0.053	0.053	–	0.352	0.361	**0.032**
	Adult_2	0.063	0.060	0.059	0.058	0.057	0.059	0.000	–	0.174	**0.003**
	Egg_1_W	0.083	0.078	0.078	0.078	0.080	0.075	0.002	0.007	–	0.089
	Egg_2_W	0.060	0.059	0.055	0.056	0.056	0.054	0.002	0.003	0.010	–

*Note*: Heatmap of pairwise *F*_ST_ values in the bottom diagonal and corresponding *p*‐values after 10,000 permutations in the top diagonal, with the ones significantly different from zero after FDR correction highlighted in boldface type. Green colours indicate low differentiation increasing towards red to indicate larger differentiation. *N* is the number of individuals in each group.

COLONY registered 31 full‐sib families with probabilities >0.913 (Table S2): 19 of them happened between individuals sampled on the farm itself (either from the same or different cages), four families were found among the escapees, while the remaining eight happened between farmed and escaped fish, thus clearly linking the escapees to the farm. Full‐sibs were not detected between eggs of farmed ancestry and the farmed fish or escapees, however, relatedness equivalent to half‐siblingship was detected between eggs and farmed fish as well as between eggs and escapees.

### 3.5 Modelling cage spawning

One of the main questions arising from the above analyses was whether the farmed cod eggs originated from spawning in the cages, where a high number of mature fish had been reported, and/or from escapees spawning in the wild. We, therefore, implemented hydrodynamic particle modelling to check if eggs of farmed ancestry could have drifted by ocean currents from the farm itself to the locations where they were sampled. Simulated particles ‘released’ at the Frosvika cod farm on February 23. at 5 m depth showed a relatively high probability of ending up in the region where most eggs of farmed origin were found on the first egg survey (February 28 to March 2), see Figure S3. This is consistent with the notion that eggs of farmed origin stemmed from spawning at or around the farm in the week preceding the survey. Simulation particles released at greater depths or other dates mostly followed a northward dispersion pattern, but there was also some southward movement. For the second survey period, the match between simulation results and egg findings was less clear, but the general dispersion pattern was similar to the first period (Figure S4).

## 4 DISCUSSION

Genetic interaction between aquaculture escapees and wild conspecifics is globally regarded as one of the main long‐term challenges to sustainable cage‐based aquaculture. While this topic has been extensively studied in salmonids (Glover et al., 2017), in marine fish, which also display the potential to ‘escape’ through within cage spawning, there is still a need for data on the type, frequency and the magnitude of this interaction (Bekkevold et al., 2006). Here, following up on a suspected escape of fish from a Norwegian cod farm, we identified sexually mature farmed escaped cod in the wild, and identified a number of eggs in the vicinity of the farm that were of farmed ancestry. While it was not possible to conclude within cage versus outside cage spawning, our work provides a rare documentation of a domesticated marine fish releasing offspring into the wild.

### 4.1 Farm of origin for the farmed cod escapees

We confirmed a farmed origin for the suspected escaped cod, matching them specifically to the local farm. In theory, these fish could have escaped from other farms that used fish from the same broodstock. However, the closest operational cod farms were located more than 200 km away from the locations where the escapees were recaptured. Moreover, we established a direct full‐ or half‐sibship relationship between escapees and fish held in cages on the farm studied. Collectively, these facts strongly point to this farm as the source. This sort of siblingship analysis between escapees and suspected farms has been used multiple times in Norway to help identify the farm of origin for Atlantic salmon escapees as part of the routine service offered by IMR to the NDF (Glover et al., 2008, 2010; Quintela et al., 2016), and works well due to the limited number of adults contributing to fish in cages. Finally, farmed cod were caught in large quantities by local commercial fishers within the study region, reinforcing our conclusion that the local farm was the source of escaped fish.

### 4.2 Origin of the farmed cod eggs

The cod eggs were not only identified as of farmed origin through STRUCTURE and genetic assignment analyses, but they were also identified as originating from fish in the farm, and/or escapees from the farm, based upon the detection of half‐sibling relationships or equivalent. This observation agrees with an earlier study that also detected genetically marked farmed cod eggs in the wild, using a genetically marked broodstock (Jørstad et al., 2008; van der Meeren et al., 2012). We found the highest proportion of farmed eggs (>80%) during the first survey, in late February‐early March, but an appreciable fraction (12%) was found to be farmed also in the second survey, 4 weeks later. The reason for the observed drop in the proportion of eggs of farmed origin between the two sampling time points may be linked with the fact that the farm in the interim was ordered by NDF to slaughter all cod in some of their cages due to a high frequency of maturation observed in the cages. Another likely explanation is that wild cod spawning is known to increase between these two time points, thus reducing the relative proportion of farmed versus wild cod eggs in the second survey when wild spawning had increased.

Among the eggs of farmed origin, no first‐sib relations were detected, in contrast to what was observed among the adults of both farmed and escaped sources. The farmed adult cod were hatched at the fry producer from what appears to be a relatively small number of parents, as indicated by the low *N*_e_ estimates for them (cf. Table 2 and discussion below). The eggs obviously belong to another generation from the farmed cod themselves and have a presumably much larger set of parents. We do not know how large a proportion of farmed cod that reached maturity and could have produced these eggs, but it could potentially be in the thousands or more. This is reflected by the relatively large estimates of effective population size for eggs of farmed origin (571 to infinity: Table 2), although the estimates are uncertain due to small sample sizes. The apparent high number of parents and the absence of full‐sib pairs among the eggs of farmed origin that were sampled (46) indicate that the majority of eggs were produced in broadcast spawning event(s).

Local coastal cod have spawning areas at several of the locations where farmed eggs were found (https://www.fiskeridir.no/English), and actual spawning of wild cod in the region is evident from the presence also of wild eggs in both egg surveys. The mixing of farmed and wild eggs at the same locations, and the identification of a single egg of potential farmed × wild origin, may indicate that escaped farmed cod are spawning at natural spawning grounds. In contrast, results from the hydrodynamic simulations demonstrated that it is possible for eggs to reach the survey locations by passive drift with ocean currents, even if spawning took place on the farm. It was, therefore, not possible to assess where the spawning of farmed cod took place, and thus, whether the presence of farmed eggs in the natural environment was directly related to the escape of farmed fish. The deficiency of farmed x wild hybrids is perhaps indicative that most or all of them originated from spawning in the farm itself, but one egg of potential hybrid origin in the first egg survey weakens this inference. Behavioural studies of courtship in farmed and wild cod indicate that hybridisation between farmed escapees and wild cod is likely, particularly between wild males and farmed females (Meager et al., 2009, 2010; Skjæraasen et al., 2010). Whether spawning of farmed cod took place in the cages or the wild, the finding of large quantities of farmed eggs supports concerns over genes of farmed origin being introduced into the local, wild cod population (Bjørn, Bøhn, et al., 2022).

### 4.3 Evolutionary and management implications

Based upon data from salmonids, the introduction of ‘farmed’ genes into wild populations has been demonstrated to alter life history characteristics (Besnier et al., 2022; Bolstad et al., 2017, 2021), and it has been demonstrated that the offspring of domesticated fish display reduced survival in the wild (McGinnity et al., 2003; Skaala et al., 2012, 2019; Sylvester et al., 2019). However, to what degree data from salmonids can be imputed to reflect the situation in marine fish such as cod is uncertain, especially as cod is still in the early stages of the domestication process (about 6 or 7 cod generations: Bjørn, Berg, et al., 2022). Little is presently known about what genetic changes have occurred in farmed cod, although the loss of genetic variability (Glover et al., 2010, 2011; this study) and genetically increased growth rate (Otterå et al., 2018) have been documented.

Assuming that the founding population for the farmed cod studied here had similar genetic variability as wild populations in the area, the loss of heterozygosity in the farmed cod was estimated to be 13%. Even more pronounced was the loss of 45% of microsatellite alleles, as assessed by the allelic richness measure. Similar losses of genetic variability have also been observed in domesticated salmonids (Skaala et al., 2004), as well as in other marine fish taken into culture (e.g. Loukovitis et al., 2015; Sawayama et al., 2019). This reduction underscores the rapid genetic change that occurs in the farmed cod stock, and the low estimate we obtained for its effective population size (*N*_e_ = 53.8, average of farmed estimates in Table 2, and referring to the parental generation) indicates that much of this change is driven by random genetic drift.

Fish farming in Norway, including cod farming, is strictly regulated and permits for farming must be obtained from the local and county municipalities after a hearing process that includes several authorities. The NDF, as a part of this process, order annual risk assessments of farming activities from the IMR (Grefsrud, Andersen, et al., 2022; Grefsrud, Bjørn, et al., 2022). Applicants of cod farming permits often stress the alleged tendency of the current domesticated stock to be less prone to escape the cages, that maturity on the farm can be controlled or hindered by the application of artificial lights, and that eggs from farmed cod will not develop into viable fry. As the present study has demonstrated, the risk of both escape and spawning of farmed cod in the cages remains and needs to be monitored closely. It is a big concern that escaped cod may go undetected and maturing cod at the farms is seen to represent a considerable risk of genetic change in wild coastal cod populations (Bjørn, Berg, et al., 2022). We note that mature cod were detected at the farm during follow‐up inspections after local fishers reported catches of mature escaped fish (NDF, 2023), leaving concerns that maturation may typically go unnoticed and be a general problem in cod aquaculture. As shown herein, and in common with other domesticated organisms, farmed cod is characterized by a marked reduction in genetic variability and this could compromise wild cod populations, should interbreeding between escapees and wild cod occur. We plan follow‐up sampling and analyses of cod in the region, in order to investigate whether any long‐term effects on the local cod population can be detected.

## CONFLICT OF INTEREST STATEMENT

The authors declare no conflicts of interest.

## Supporting information


Data S1:

## Data Availability

The genotype raw data and metadata used in this study are available at the public repository: https://imr.brage.unit.no/imr‐xmlui/handle/11250/3121419.

## References

[eva13688-bib-0001] Ådlandsvik, B. , & Sundby, S. (1994). Modelling the transport of cod larvae from the Lofoten area. ICES Marine Science Symposia, 198, 379–392.

[eva13688-bib-0002] Albretsen, J. , Sperrevik, A. K. , Staalstrøm, A. , Sandvik, A. D. , Vikebø, F. , & Asplin, L. (2011). NorKyst‐800 report No. 1: User manual and technical descriptions. Fisken og Havet, 2–11, 1–46.

[eva13688-bib-0003] Alvanou, M. V. , Gkagkavouzis, K. , Karaiskou, N. , Feidantsis, K. , Lattos, A. , Michaelidis, B. , Theodorou, J. A. , Batargias, C. , Triantafyllidis, A. , & Giantsis, I. A. (2023). Mediterranean aquaculture and genetic pollution: A review combined with data from a fish farm evaluating the ecological risks of finfish escapes. Journal of Marine Science and Engineering, 11, 1405.

[eva13688-bib-0004] Asplin, L. , Albretsen, J. , Johnsen, I. A. , & Sandvik, A. D. (2020). The hydrodynamic foundation for salmon lice dispersion modeling along the Norwegian coast. Ocean Dynamics, 70, 1151–1167.

[eva13688-bib-0005] Bekkevold, D. , Hansen, M. M. , & Nielsen, E. E. (2006). Genetic impact of gadoid culture on wild fish populations: Predictions, lessons from salmonids, and possibilities for minimizing adverse effects. ICES Journal of Marine Science, 63, 198–208.

[eva13688-bib-0006] Benjamini, Y. , & Hochberg, Y. (1995). Controlling the false discovery rate: A practical and powerful approach to multiple testing. Journal of the Royal Statistical Society: Series B: Methodological, 57, 289–300.

[eva13688-bib-0007] Besnier, F. , Ayllon, F. , Skaala, O. , Solberg, M. F. , Fjeldheim, P. T. , Anderson, K. , Knutar, S. , & Glover, K. A. (2022). Introgression of domesticated salmon changes life history and phenology of a wild salmon population. Evolutionary Applications, 15, 853–864. 35603027 10.1111/eva.13375 PMC9108307

[eva13688-bib-0008] Bjørn, P. A. , Berg, E. , Bøhn, T. , Espeland, S. H. , Glover, K. , Jorde, P. E. , Karlsen, Ø. , van der Meeren, T. , Meier, S. , Moland, E. , Myksvoll, M. S. , Sandlund, N. , Sævik, P. N. , Karlsbakk, E. , Søther, B.‐S. , & Sætra, I. M. (2022). Effekter av torskeoppdrett i åpne merder på ville torskebestander [In Norwegian] . In: Grefsrud, Bjørn, et al. pp. 314–358.

[eva13688-bib-0009] Bjørn, P. A. , Bøhn, T. , van der Meeren, T. , Meier, S. , Jorde, P. E. , Sandlund, N. , Sævik, P. N. , Espeland, S. H. , Berg, E. , & Karlsbakk, E. (2022). Risiko knyttet til effekter av torskeoppdrett i åpne merder på ville torskebestander [in Norwegian] . In: Grefsrud, Andersen, et al., pp. 184–208.

[eva13688-bib-0010] Bolstad, G. H. , Hindar, K. , Robertsen, G. , Jonsson, B. , Sægrov, H. , Diserud, O. H. , Fiske, P. , Jensen, A. J. , Urdal, K. , Næsje, T. F. , Barlaup, B. T. , Florø‐Larsen, B. , Lo, H. , Niemelä, E. , & Karlsson, S. (2017). Gene flow from domesticated escapees alters the life history of wild Atlantic salmon. Nature Ecology & Evolution, 1, 124. 28812692 10.1038/s41559-017-0124

[eva13688-bib-0011] Bolstad, G. H. , Karlsson, S. , Hagen, I. J. , Fiske, P. , Urdal, K. , Sægrov, H. , Florø‐Larsen, B. , Sollien, V. P. , Østborg, G. , Diserud, O. H. , Jensen, A. J. , & Hindar, K. (2021). Introgression from farmed escapees affects the full life cycle of wild Atlantic salmon. Science Advances, 7, eabj3397. 34936452 10.1126/sciadv.abj3397 PMC8694624

[eva13688-bib-0012] Bostock, J. , McAndrew, B. , Richards, R. , Jauncey, K. , Telfer, T. , Lorenzen, K. , Little, D. , Ross, L. , Handisyde, N. , Gatward, I. , & Corner, R. (2010). Aquaculture: Global status and trends. Philosophical Transactions of the Royal Society of London, Series B: Biological Sciences, 365, 2897–2912. 20713392 10.1098/rstb.2010.0170 PMC2935128

[eva13688-bib-0013] Bradbury, I. R. , Burgetz, I. , Coulson, M. W. , Verspoor, E. , Gilbey, J. , Lehnert, S. J. , Kess, T. , Cross, T. F. , Vasemägi, A. , Solberg, M. F. , Fleming, I. A. , & McGinnity, P. (2020). Beyond hybridization: The genetic impacts of non‐reproductive ecological interactions of salmon aquaculture on wild populations. Aquaculture Environment Interactions, 12, 429–445.

[eva13688-bib-0014] Cao, L. , Wang, W. , Yang, Y. , Yang, C. , Yuan, Z. , Xiong, S. , & Diana, J. (2007). Environmental impact of aquaculture and countermeasures to aquaculture pollution in China. Environmental Science and Pollution Research, 14, 452–462. 18062476 10.1065/espr2007.05.426

[eva13688-bib-0015] Dalsøren, S. B. , Albretsen, J. , & Asplin, L. (2020). New validation method for hydrodynamic fjord models applied in the Hardangerfjord, Norway. Estuarine, Coastal and Shelf Science, 246, 107028.

[eva13688-bib-0016] Damsgård, B. , Hoy, E. , Uglem, I. , Hedger, R. D. , Izquierdo‐Gomez, D. , & Bjørn, P. A. (2012). Net‐biting and escape behaviour in farmed Atlantic cod *Gadus morhua*: Effects of feed stimulants and net traits. Aquaculture Environment Interactions, 3, 1–9.

[eva13688-bib-0017] Dieringer, D. , & Schlötterer, C. (2003). Microsatellite analyser (MSA): A platform independent analysis tool for large microsatellite data sets. Molecular Ecology Notes, 3, 167–169.

[eva13688-bib-0018] Duval, E. , Skaala, Ø. , Quintela, M. , Dahle, G. , Delaval, A. , Wennevik, A. , Glover, K. A. , & Hansen, M. M. (2021). Long‐term monitoring of a brown trout (*Salmo trutta*) population reveals kin‐associated migration patterns and contributions by resident trout to the anadromous run. BMC Ecology and Evolution, 21, 143. 34256705 10.1186/s12862-021-01876-9 PMC8276402

[eva13688-bib-0019] Edwards, P. (2015). Aquaculture environment interactions: Past, present and likely future trends. Aquaculture, 447, 2–14.

[eva13688-bib-0020] Excoffier, L. , Laval, G. , & Schneider, S. (2005). Arlequin ver. 3.0: An integrated software package for population genetics data analysis. Evolutionary Bioinformatics Online, 1, 47–50. PMC2658868 19325852

[eva13688-bib-0021] Glover, K. A. (2010). Forensic identification of fish farm escapees: The Norwegian experience. Aquaculture Environment Interactions, 1, 1–10.

[eva13688-bib-0022] Glover, K. A. , Dahle, G. , & Jørstad, K. E. (2011). Genetic identification of farmed and wild Atlantic cod, *Gadus morhua*, in coastal Norway. ICES Journal of Marine Science, 68, 901–910.

[eva13688-bib-0023] Glover, K. A. , Dahle, G. , Westgaard, J.‐I. , Johansen, T. , Knutsen, H. , & Jørstad, K. E. (2010). Genetic diversity within and among strains of farmed Atlantic cod (*Gadus morhua*): A proof‐of‐concept study for the identification of escapees. Animal Genetics, 41, 515–522. 20331613 10.1111/j.1365-2052.2010.02025.x PMC3068200

[eva13688-bib-0024] Glover, K. A. , Skilbrei, O. T. , & Skaala, Ø. (2008). Genetic assignment identifies farm of origin for Atlantic salmon *Salmo salar* escapees in a Norwegian fjord. ICES Journal of Marine Science, 65, 912–920.

[eva13688-bib-0025] Glover, K. A. , Solberg, M. F. , McGinnity, P. , Hindar, K. , Verspoor, E. , Coulson, M. W. , Hansen, M. M. , Araki, H. , Skaala, Ø. , & Svåsand, T. (2017). Half a century of genetic interaction between farmed and wild Atlantic salmon: Status of knowledge and unanswered questions. Fish and Fisheries, 18, 890–927.

[eva13688-bib-0026] Grefsrud, E. S. , Andersen, L. B. , Bjørn, P. A. , Grøsvik, B. E. , Hansen, P. K. , Husa, V. , Karlsen, Ø. , Kvamme, B. O. , Samuelsen, O. , Sandlund, N. , Solberg, M. F. , & Stien, L. H. (2022). Risikorapport norsk fiskeoppdrett 2022 – risikovurdering [in Norwegian] . Rapport Fra Havforskningen 2022‐12.

[eva13688-bib-0027] Grefsrud, E. S. , Bjørn, P. A. , Grøsvik, B. E. , Hansen, P. K. , Husa, V. , Karlsen, Ø. , Kvamme, B. O. , Samuelsen, O. , Sandlund, N. , Solberg, M. F. , & Stien, L. H. (2022). Risikorapport norsk fiskeoppdrett 2022 – kunnskapsstatus [in Norwegian] . Rapport Fra Havforskningen 2022‐13.

[eva13688-bib-0028] Hansen, L. A. , Dale, T. , Damsgard, B. , Uglem, I. , Aas, K. , & Bjørn, P. A. (2008). Escape‐related behaviour of Atlantic cod, *Gadus morhua* L., in a simulated farm situation. Aquaculture Research, 40, 26–34.

[eva13688-bib-0029] Jakobsson, M. , & Rosenberg, N. A. (2007). CLUMPP: A cluster matching and permutation program for dealing with label switching and multimodality in analysis of population structure. Bioinformatics, 23, 1801–1806. 17485429 10.1093/bioinformatics/btm233

[eva13688-bib-0030] Jensen, O. , Dempster, T. , Thorstad, E. B. , Uglem, I. , & Fredheim, A. (2010). Escapes of fishes from Norwegian sea‐cage aquaculture: Causes, consequences and prevention. Aquaculture Environment Interactions, 1, 71–83.

[eva13688-bib-0031] Jombart, T. (2008). Adegenet: A R package for the multivariate analysis of genetic markers. Bioinformatics, 24, 1403–1405. 18397895 10.1093/bioinformatics/btn129

[eva13688-bib-0032] Jombart, T. , & Collins, C. (2015). A tutorial for Discriminant Analysis of Principal Components (DAPC) using adegenet 2.0.0. Imperial College London, MRC Centre for Outbreak Analysis and Modelling.

[eva13688-bib-0033] Jombart, T. , Devillard, S. , & Balloux, F. (2010). Discriminant analysis of principal components: A new method for the analysis of genetically structured populations. BMC Genetics, 11, 94. 20950446 10.1186/1471-2156-11-94 PMC2973851

[eva13688-bib-0034] Jones, O. R. , & Wang, J. (2010). COLONY: A program for parentage and sibship inference from multilocus genotype data. Molecular Ecology Resources, 10, 551–555. 21565056 10.1111/j.1755-0998.2009.02787.x

[eva13688-bib-0035] Jørstad, K. E. , Otterå, H. , van der Meeren, T. , Dahle, G. , Paulsen, O. I. , Bagge, G. , & Svåsand, T. (2014). Genetic marking of farmed Atlantic cod (*Gadus morhua* L.) and detection of escapes from a commercial cod farm. ICES Journal of Marine Science, 71, 574–584.

[eva13688-bib-0036] Jørstad, K. E. , van der Meeren, T. , Paulsen, O. I. , Thomsen, T. , Thorsen, A. , & Svåsand, T. (2008). “escapes” of eggs from farmed cod spawning in net pens: Recruitment to wild stocks. Reviews in Fisheries Science, 16, 285–295.

[eva13688-bib-0037] Loukovitis, D. , Ioannidi, B. , Chatziplis, D. , Kotoulas, G. , Magoulas, A. , & Tsigenopoulos, C. S. (2015). Loss of genetic variation in Greek hatchery populations of the European sea bass (*Dicentrarchus labrax* L.) as revealed by microsatellite DNA analysis. Mediterranean Marince Science, 16, 197–200.

[eva13688-bib-0038] Mateos‐Rivera, A. , Skern‐Mauritzen, R. , Dahle, G. , Sundby, S. , Mozfar, B. , Thorsen, A. , Wehde, H. , & Krafft, B. A. (2020). Comparison of visual and molecular taxonomic methods to identify ichthyoplankton in the North Sea. Limnology and Oceanography: Methods, 18, 599–605.

[eva13688-bib-0039] McGinnity, P. , Prodöhl, P. , Ferguson, A. , Hynes, R. , Maoiléidigh, N. , Baker, N. , Cotter, D. , O'Hea, B. , Cooke, D. , Rogan, G. , Taggart, J. , & Cross, T. (2003). Fitness reduction and potential extinction of wild populations of Atlantic salmon, *Salmo salar*, as a result of interactions with escaped farm salmon. Proceedings of the Royal Society B: Biological Sciences, 270, 2443–2450. 10.1098/rspb.2003.2520 PMC1691531 14667333

[eva13688-bib-0040] Meager, J. J. , Skjæraasen, J. E. , Fernö, A. , Karlsen, Ø. , Løkkeborg, S. , Michalsen, K. , & Utskot, S. O. (2009). Vertical dynamics and reproductive behaviour of farmed and wild Atlantic cod *Gadus morhua* . Marine Ecology Progress Series, 389, 233–243.

[eva13688-bib-0041] Meager, J. J. , Skjæraasen, J. E. , Fernö, A. , & Løkkeborg, S. (2010). Reproductive interactions between fugitive farmed and wild Atlantic cod (*Gadus morhua*) in the field. Canadian Journal of Fisheries and Aquatic Sciences, 67, 1221–1231.

[eva13688-bib-0042] Miller, J. M. , Cullingham, C. I. , & Peery, R. M. (2020). The influence of a priori grouping on inference of genetic clusters: Simulation study and literature review of the DAPC method. Heredity, 125, 269–280. 32753664 10.1038/s41437-020-0348-2 PMC7553915

[eva13688-bib-0043] Myers, R. A. , Hutchings, J. A. , & Barrowman, N. J. (1997). Why do fish stocks collapse? The example of cod in Atlantic Canada. Ecological Applications, 7, 91–106.

[eva13688-bib-0044] Naylor, R. L. , Hardy, R. W. , Buschmann, A. H. , Bush, S. R. , Cao, L. , Klinger, D. H. , Little, D. C. , Lubchenco, J. , Shumway, S. E. , & Troell, M. (2021). A 20‐year retrospective review of global aquaculture. Nature, 591, 551–563. 33762770 10.1038/s41586-021-03308-6

[eva13688-bib-0045] NDF . (2023). Fiskeridirektoratets oppfølging etter funn av rømt og moden oppdrettstorsk i Meløy kommune, Nordland. [In Norwegian] Fiskeridirektoratet, oktober 2023 .

[eva13688-bib-0046] Otterå, H. , Heino, M. , Sorvik, A. G. E. , Svåsand, T. , Karlsen, Ø. , Thorsen, A. , & Glover, K. A. (2018). Growth of wild and domesticated Atlantic cod *Gadus morhua* reared under semi‐commercial conditions. Aquaculture Environment Interactions, 10, 187–200.

[eva13688-bib-0047] Peakall, R. , & Smouse, P. E. (2006). GenAlEx 6: Genetic analysis in excel. Population genetic software for teaching and research. Molecular Ecology Notes, 6, 288–295. 10.1093/bioinformatics/bts460 PMC3463245 22820204

[eva13688-bib-0048] Pershing, A. J. , Alexander, M. A. , Hernandez, C. M. , Kerr, L. A. , le Bris, A. , Mills, K. E. , Nye, J. A. , Record, N. R. , Scannell, H. A. , Scott, J. D. , Sherwood, G. D. , & Thomas, A. C. (2015). Slow adaptation in the face of rapid warming leads to collapse of the Gulf of Maine cod fishery. Science, 350, 809–812. 26516197 10.1126/science.aac9819

[eva13688-bib-0049] Piry, S. , Alapetite, A. , Cornuet, J.‐M. , Paetkau, D. , Baudouin, L. , & Estoup, A. (2004). GENECLASS2: A software for genetic assignment and first‐generation migrant detection. Journal of Heredity, 95, 536–539. 15475402 10.1093/jhered/esh074

[eva13688-bib-0050] Pritchard, J. K. , Stephens, M. , & Donnelly, P. (2000). Inference of population structure using multilocus genotype data. Genetics, 155, 945–959. 10835412 10.1093/genetics/155.2.945 PMC1461096

[eva13688-bib-0051] Puvanendran, V. , Mortensen, A. , Johansen, L. H. , Kettunen, A. , Hansen, Ø. J. , Henriksen, E. , & Heide, M. (2022). Development of cod farming in Norway: Past and current biological and market status and future prospects and directions. Reviews in Aquaculture, 14, 308–342.

[eva13688-bib-0052] Quintela, M. , Wennevik, V. , Sørvik, A. G. E. , Skaala, Ø. , Skilbrei, O. T. , Urdal, K. , Barlaup, B. T. , & Glover, K. A. (2016). Siblingship tests connect two seemingly independent farmed Atlantic salmon escape events. Aquaculture Environment Interactions, 8, 497–509.

[eva13688-bib-0053] R Core Team . (2023). R: A language and environment for statistical computing. R Foundation for Statistical Computing. https://www.R‐project.org/

[eva13688-bib-0054] Rannala, B. , & Mountain, J. L. (1997). Detecting immigration by using multilocus genotypes. Proceedings of the National Academy of Sciences of the United States of America, 94, 9197–9201. 9256459 10.1073/pnas.94.17.9197 PMC23111

[eva13688-bib-0055] Röhrs, J. , Christensen, K. H. , Vikebø, F. , Sundby, S. , Saetra, Ø. , & Broström, G. (2014). Wave‐induced transport and vertical mixing of pelagic eggs and larvae. Limnology and Oceanography, 59, 1213–1227.

[eva13688-bib-0056] Rousset, F. (2008). GENEPOP'007: A complete re‐implementation of the genepop software for windows and Linux. Molecular Ecology Resources, 8, 103–106. 21585727 10.1111/j.1471-8286.2007.01931.x

[eva13688-bib-0057] Sawayama, E. , Nakao, H. , Kobayashi, W. , Minami, T. , & Takagi, M. (2019). Identification and quantification of farmed red sea bream escapees from a large aquaculture area in Japan using microsatellite DNA markers. Aquatic Living Resources, 32, 26.

[eva13688-bib-0058] Shchepetkin, A. F. , & McWilliams, J. C. (2005). The regional oceanic modeling system (ROMS): A split‐explicit, free‐surface, topography‐following‐coordinate oceanic model. Ocean Modelling, 9, 347–404.

[eva13688-bib-0059] Skaala, Ø. , Besnier, F. , Borgstrøm, R. , Barlaup, B. T. , Sørvik, A. G. , Normann, E. , Østebø, B. I. , Hansen, M. M. , & Glover, K. A. (2019). An extensive commongarden study with domesticated and wild Atlantic salmon in the wild reveals impact on smolt production and shifts in fitness traits. Evolutionary Applications, 12, 1001–1016. 31080511 10.1111/eva.12777 PMC6503829

[eva13688-bib-0060] Skaala, Ø. , Glover, K. A. , Barlaup, B. T. , Svåsand, T. , Besnier, F. , Hansen, M. M. , & Borgstrøm, R. (2012). Performance of farmed, hybrid, and wild Atlantic salmon (*Salmo salar*) families in a natural river environment. Canadian Journal of Fisheries and Aquatic Sciences, 69, 1994–2006.

[eva13688-bib-0061] Skaala, Ø. , Høyheim, B. , Glover, K. A. , & Dahle, G. (2004). Microsatellite analysis in domesticated and wild Atlantic salmon (*Salmo salar* L): Allelic diversity and identification of individuals. Aquaculture, 240, 131–143.

[eva13688-bib-0062] Skjæraasen, J. E. , Meager, J. J. , Karlsen, Ø. , Mayer, I. , Dahle, G. , Rudolfsen, G. , & Fernö, A. (2010). Mating competition between farmed and wild cod *Gadus morhua* . Marine Ecology Progress Series, 412, 247–258.

[eva13688-bib-0063] Soto, D. , Arismendi, I. , Olivos, J. A. , Canales‐Aguirre, C. B. , Leon‐Muñoz, J. , Niklitschek, E. J. , Sepúlveda, M. , Paredes, F. , Gomez‐Uchida, D. , & Soria‐Galvarro, Y. (2023). Environmental risk assessment of non‐native salmonid escapes from net pens in the Chilean Patagonia. Reviews in Aquaculture, 15, 198–219.

[eva13688-bib-0064] Sylvester, E. V. A. , Wringe, B. F. , Duffy, S. J. , Hamilton, L. C. , Fleming, I. A. , Castellani, M. , Bentzen, P. , & Bradbury, I. R. (2019). Estimating the relative fitness of escaped farmed salmon offspring in the wild and modelling the consequences of invasion for wild populations. Evolutionary Applications, 12, 705–717. 30976304 10.1111/eva.12746 PMC6439497

[eva13688-bib-0065] Taranger, G. L. , Karlsen, O. , Bannister, R. J. , Glover, K. A. , Husa, V. , Karlsbakk, E. , Kvamme, B. O. , Boxaspen, K. K. , Bjørn, P. A. , Finstad, B. , Madhun, A. S. , Morton, H. C. , & Svåsand, T. (2015). Risk assessment of the environmental impact of Norwegian Atlantic salmon farming. ICES Journal of Marine Science, 72, 997–1021.

[eva13688-bib-0066] Teletchea, F. , & Fontaine, P. (2014). Levels of domestication in fish: Implications for the sustainable future of aquaculture. Fish and Fisheries, 15, 181–195.

[eva13688-bib-0067] van der Meeren, T. , Jørstad, K. E. , Paulsen, O. I. , & Dahle, G. (2012). Offspring from farmed cod (Gadus morhua L.) spawning in net pens: Documentation of larval survival, recruitment to spawning stock, and successful reproduction . Ices CM 2012/P:11. 12 pp. (in mimeo). https://www.ices.dk/sites/pub/CM%20Doccuments/CM‐2012/P/P1112.pdf

[eva13688-bib-0068] Varne, R. , Kunz, K. L. , Johansen, T. , Westgaard, J. I. , Uglem, I. , & Mork, J. (2015). Farmed cod escapees and net‐pen spawning left no clear genetic footprint in the local wild cod population. Aquaculture Environment Interactions, 7, 253–266.

[eva13688-bib-0069] Waples, R. S. , & Do, C. (2008). LDNE: A program for estimating effective population size from data on linkage disequilibrium. Molecular Ecology Resources, 8, 753–756. 21585883 10.1111/j.1755-0998.2007.02061.x

[eva13688-bib-0070] Weir, B. S. , & Cockerham, C. C. (1984). Estimating F‐statistics for the analysis of population structure. Evolution, 38, 1358–1370. 28563791 10.1111/j.1558-5646.1984.tb05657.x

