# Supplementary material for: Genetic analyses verify sexually mature escaped farmed Atlantic cod and farmed cod eggs in the natural environment
Source: Evol Appl. 2024 Apr 17;17(4):e13688. doi: 10.1111/eva.13688 (PMC11022607; doi:10.1111/eva.13688)
Supplement: Supplementary file 1 — Data S1: [file EVA-17-e13688-s001.docx]

**Supporting Information**

**Table S1**. Information for PCR reactions. Multiplex, locus, primer volume from a stock concentration of 10 µL, PCR conditions for multiplex, reference for locus primers.

| **Multiplex** | **Locus** | **Volume (****µL)** | **Initial denaturation** | **N cycles** | **Denaturation** | **Annealing** | **Extension** | **Final elongation** | **Ref. locus primers** |
| --- | --- | --- | --- | --- | --- | --- | --- | --- | --- |
| 1 | Gmo8 | 0.08 | 5 min at 95°C | 30 | 95°C - 30 sec | 56°C - 2 min | 72°C - 1 min | 72°C - 10 min | 1 |
| 1 | Gmo19 | 0.32 | 5 min at 95°C | 30 | 95°C - 30 sec | 56°C - 2 min | 72°C - 1 min | 72°C - 10 min | 1 |
| 1 | Gmo35 | 0.4 | 5 min at 95°C | 30 | 95°C - 30 sec | 56°C - 2 min | 72°C - 1 min | 72°C - 10 min | 1 |
| 1 | Gmo37 | 2 | 5 min at 95°C | 30 | 95°C - 30 sec | 56°C - 2 min | 72°C - 1 min | 72°C - 10 min | 1 |
| 1 | Tch11 | 0.64 | 5 min at 95°C | 30 | 95°C - 30 sec | 56°C - 2 min | 72°C - 1 min | 72°C - 10 min | 2 |
| 2 | Gmo2 | 0.9 | 5 min at 95°C | 30 | 95°C - 30 sec | 56°C - 2 min | 72°C - 1 min | 72°C - 10 min | 3 |
| 2 | Gmo132 | 1.1 | 5 min at 95°C | 30 | 95°C - 30 sec | 56°C - 2 min | 72°C - 1 min | 72°C - 10 min | 3 |
| 2 | Gmo3 | 0.16 | 5 min at 95°C | 30 | 95°C - 30 sec | 56°C - 2 min | 72°C - 1 min | 72°C - 10 min | 1 |
| 2 | Gmo34 | 0.16 | 5 min at 95°C | 30 | 95°C - 30 sec | 56°C - 2 min | 72°C - 1 min | 72°C - 10 min | 1 |
| 2 | Tch13 | 1.2 | 5 min at 95°C | 30 | 95°C - 30 sec | 56°C - 2 min | 72°C - 1 min | 72°C - 10 min | 2 |
| 3 | GmoC78 | 0.075 | 5 min at 95°C | 26 | 95°C - 30 sec | 57°C - 90 sec | 72°C - 1 min | 60°C - 30 min | 4 |
| 3 | GmoC80 | 0.05 | 5 min at 95°C | 26 | 95°C - 30 sec | 57°C - 90 sec | 72°C - 1 min | 60°C - 30 min | 4 |
| 3 | GmoC83 | 0.05 | 5 min at 95°C | 26 | 95°C - 30 sec | 57°C - 90 sec | 72°C - 1 min | 60°C - 30 min | 4 |
| 3 | GmoC303 | 0.075 | 5 min at 95°C | 26 | 95°C - 30 sec | 57°C - 90 sec | 72°C - 1 min | 60°C - 30 min |  |
| 3 | GmoC305 | 0.039 | 5 min at 95°C | 26 | 95°C - 30 sec | 57°C - 90 sec | 72°C - 1 min | 60°C - 30 min |  |
| 4 | GmoG12 | 0.075 | 5 min at 95°C | 26 | 95°C - 30 sec | 57°C - 90 sec | 72°C - 1 min | 60°C - 30 min | 5 |
| 4 | GmoC272, | 0.15 | 5 min at 95°C | 26 | 95°C - 30 sec | 57°C - 90 sec | 72°C - 1 min | 60°C - 30 min | 6 |
| 4 | GmoC274 | 0.05 | 5 min at 95°C | 26 | 95°C - 30 sec | 57°C - 90 sec | 72°C - 1 min | 60°C - 30 min | 6 |
| 4 | GmoC127 | 0.125 | 5 min at 95°C | 26 | 95°C - 30 sec | 57°C - 90 sec | 72°C - 1 min | 60°C - 30 min |  |
| 4 | GmoC343 | 0.15 | 5 min at 95°C | 26 | 95°C - 30 sec | 57°C - 90 sec | 72°C - 1 min | 60°C - 30 min |  |
| 4 | GMOG25a | 0.15 | 5 min at 95°C | 26 | 95°C - 30 sec | 57°C - 90 sec | 72°C - 1 min | 60°C - 30 min |  |

References: ^1^ Miller *et al.* (2000); ^2^ O'Reilly *et al.* 2000; ^3^ Brooker *et al.* (1994); ^4^ Stenvik *et al.* 2006; ^5^ Wesmajervi *et al.* (2007); ^6^ Delghandi *et al.* 2009)

**Table** **S2**. COLONY: List of fullsib families with the highest probabilities and their respective members. RF depicts escapees whereas M depicts farmed individuals.

| **Family** | **Prob** | **Member1** | **Member2** | **Member3** | **Member4** | **Member5** | **Member6** | **Member7** | **Member8** | **Member9** | **Member10** | **Member11** | **Member12** | **Member13** | **Member14** |
| --- | --- | --- | --- | --- | --- | --- | --- | --- | --- | --- | --- | --- | --- | --- | --- |
| Family_1 | 1.000 | M1_29 | M1_41 | M1_45 | M2_19 | M4_11 | M4_29 | M4_34 | RF_7 | RF_25 | RF_32 | RF_45 | RF_58 | RF_67 | RF_75 |
| Family_2 | 1.000 | M2_16 | RF_4 | RF_10 | RF_27 | RF_77 |  |  |  |  |  |  |  |  |  |
| Family_3 | 0.998 | M1_39 | RF_53 | RF_72 |  |  |  |  |  |  |  |  |  |  |  |
| Family_4 | 0.957 | M1_34 | RF_21 | RF_22 |  |  |  |  |  |  |  |  |  |  |  |
| Family_5 | 0.998 | M4_38 | RF_47 |  |  |  |  |  |  |  |  |  |  |  |  |
| Family_6 | 0.997 | M4_45 | RF_76 |  |  |  |  |  |  |  |  |  |  |  |  |
| Family_7 | 0.994 | M4_36 | RF_60 |  |  |  |  |  |  |  |  |  |  |  |  |
| Family_8 | 0.969 | M2_17 | RF_66 |  |  |  |  |  |  |  |  |  |  |  |  |
| Family_9 | 1.000 | M2_5 | M2_6 | M2_8 | M2_11 | M2_42 |  |  |  |  |  |  |  |  |  |
| Family_10 | 1.000 | M1_11 | M2_13 | M2_45 | M4_12 | M4_17 |  |  |  |  |  |  |  |  |  |
| Family_11 | 1.000 | M1_38 | M2_29 | M4_33 | M4_43 |  |  |  |  |  |  |  |  |  |  |
| Family_12 | 0.999 | M1_13 | M1_20 | M2_21 | M4_27 |  |  |  |  |  |  |  |  |  |  |
| Family_13 | 0.998 | M4_13 | M4_22 | M4_26 | M4_48 |  |  |  |  |  |  |  |  |  |  |
| Family_14 | 0.997 | M1_24 | M1_43 | M4_4 |  |  |  |  |  |  |  |  |  |  |  |
| Family_15 | 1.000 | M4_7 | M4_8 | M4_40 |  |  |  |  |  |  |  |  |  |  |  |
| Family_16 | 0.979 | M1_6 | M2_12 | M2_32 |  |  |  |  |  |  |  |  |  |  |  |
| Family_17 | 0.998 | M1_32 | M2_9 |  |  |  |  |  |  |  |  |  |  |  |  |
| Family_18 | 0.997 | M1_37 | M4_21 |  |  |  |  |  |  |  |  |  |  |  |  |
| Family_19 | 0.997 | M1_40 | M2_22 |  |  |  |  |  |  |  |  |  |  |  |  |
| Family_20 | 0.996 | M1_14 | M1_21 |  |  |  |  |  |  |  |  |  |  |  |  |
| Family_21 | 0.958 | M1_7 | M2_14 |  |  |  |  |  |  |  |  |  |  |  |  |
| Family_22 | 0.942 | M1_1 | M1_18 |  |  |  |  |  |  |  |  |  |  |  |  |
| Family_23 | 0.942 | M1_28 | M2_26 |  |  |  |  |  |  |  |  |  |  |  |  |
| Family_24 | 0.913 | M1_26 | M2_40 |  |  |  |  |  |  |  |  |  |  |  |  |
| Family_25 | 0.999 | M1_46 | M2_30 |  |  |  |  |  |  |  |  |  |  |  |  |
| Family_26 | 0.994 | M4_5 | M4_9 |  |  |  |  |  |  |  |  |  |  |  |  |
| Family_27 | 0.988 | M1_4 | M4_28 |  |  |  |  |  |  |  |  |  |  |  |  |
| Family_28 | 0.999 | RF_18 | RF_23 | RF_24 | RF_31 | RF_78 |  |  |  |  |  |  |  |  |  |
| Family_29 | 0.999 | RF_65 | RF_73 |  |  |  |  |  |  |  |  |  |  |  |  |
| Family_30 | 0.994 | RF_39 | RF_56 |  |  |  |  |  |  |  |  |  |  |  |  |
| Family_31 | 0.989 | RF_15 | RF_71 |  |  |  |  |  |  |  |  |  |  |  |  |


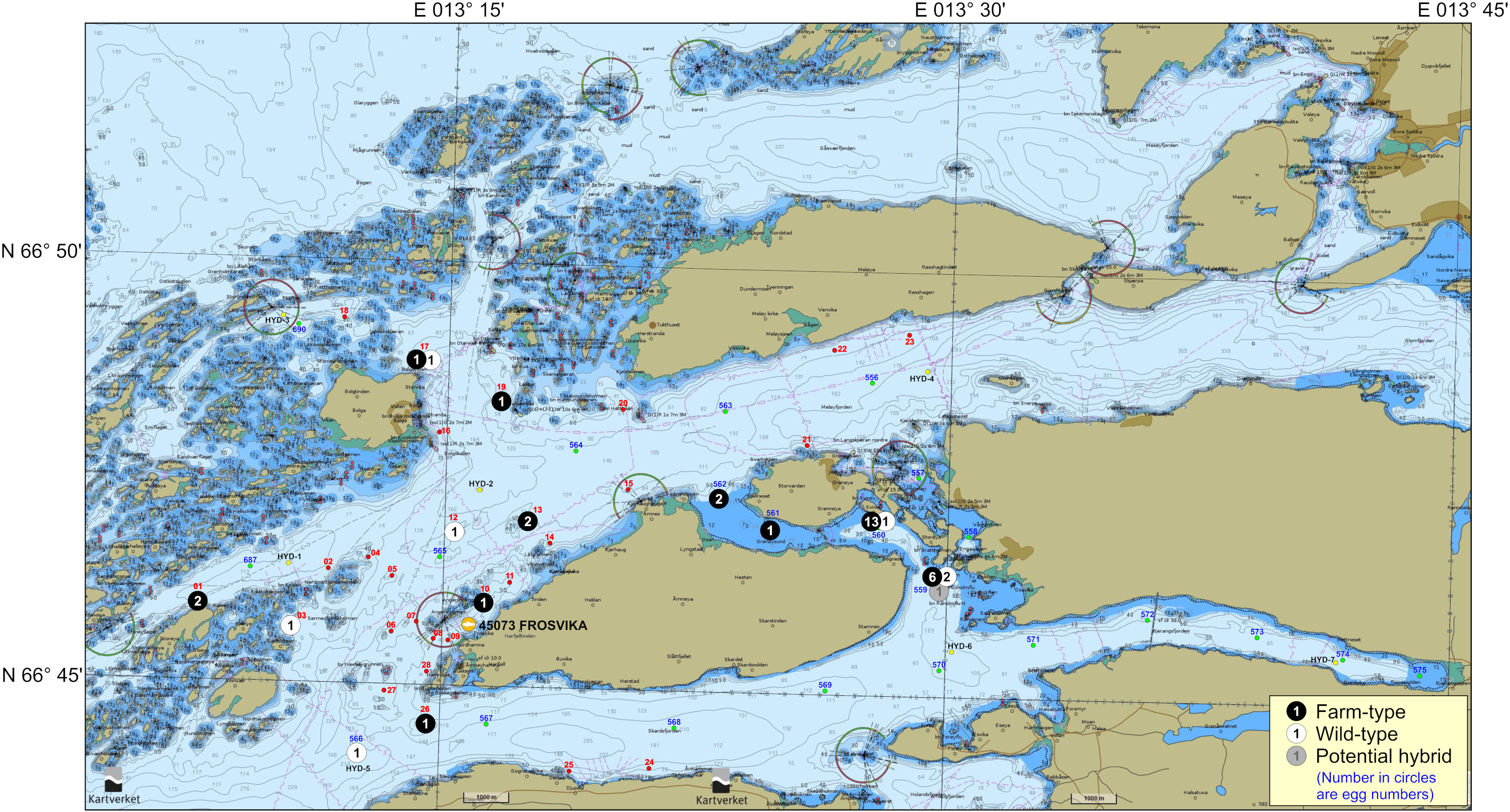


**Figure S1**. Map over study area depicting results of first egg survey (during 28. February to 2. March 2023) with eggs classified genetically to wild (white circles), farmed (black) or potential hybrid (gray) origin. Red numbers are egg haul stations, and numbers within circles represent number of eggs genetically determined to type. Coordinates are in EU89. (Source: ©norgeskart.no)


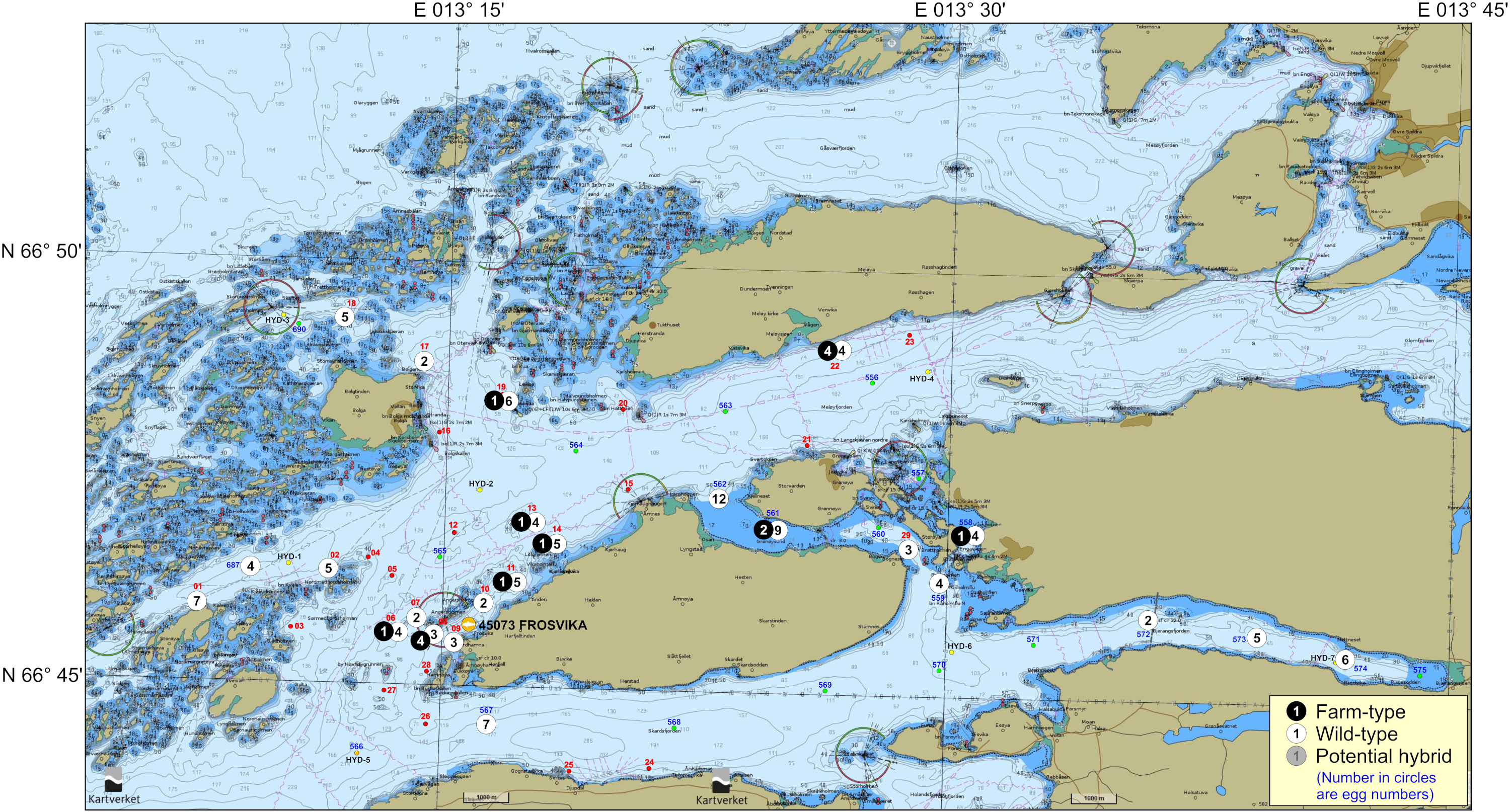


**Figure S2**. As in Fig. S1 but for the second egg survey (28. to 30. March 2023). (Source: ©norgeskart.no)


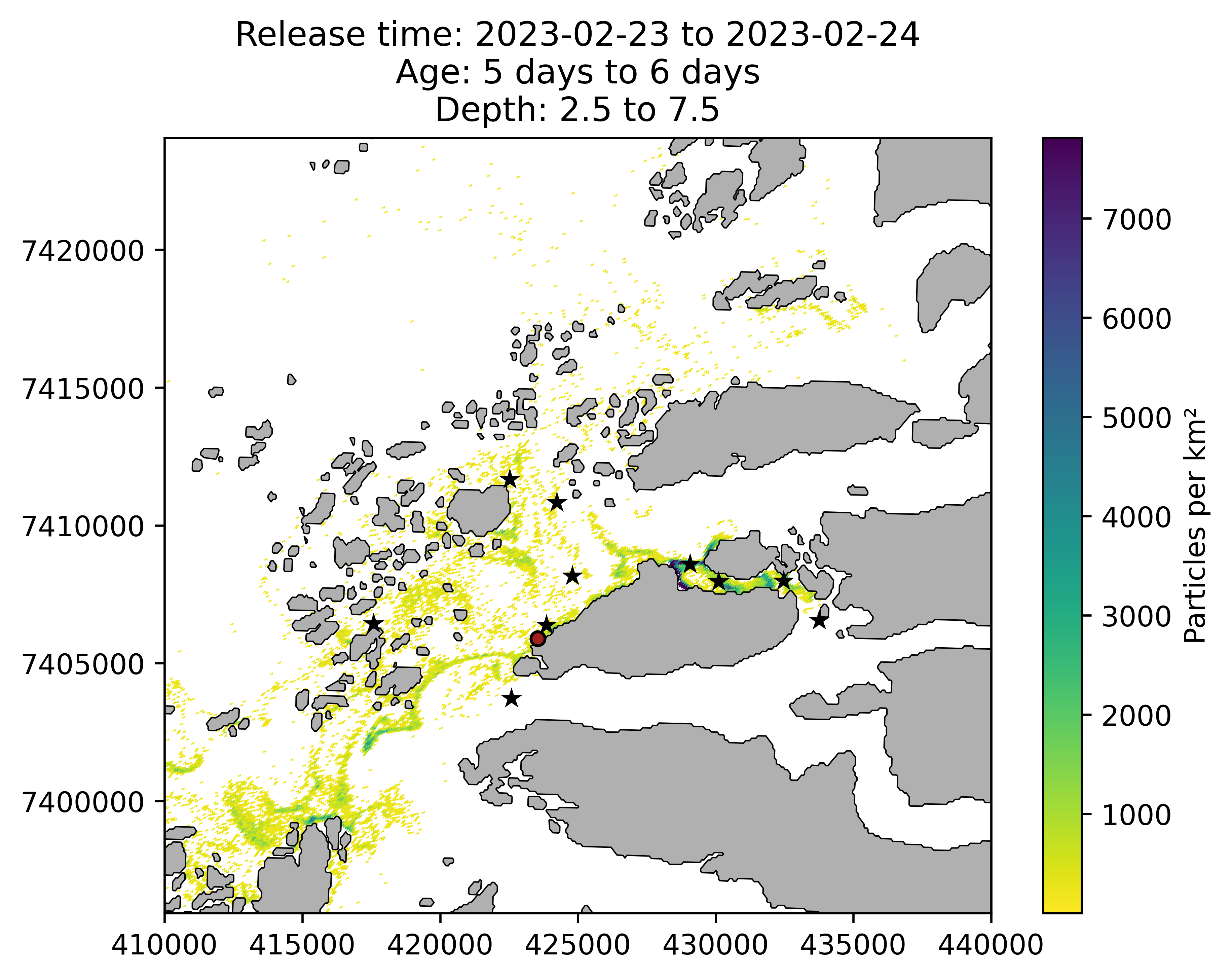


**Figure S3:** Dispersion pattern of particles released from the Frosvika cod farm (red dot) at 5 meter depth 5-6 days prior to the first egg cruise. Note: high concentrations are observed in the region where most eggs of farmed origin were found (black stars). Coordinates are in UTM33.


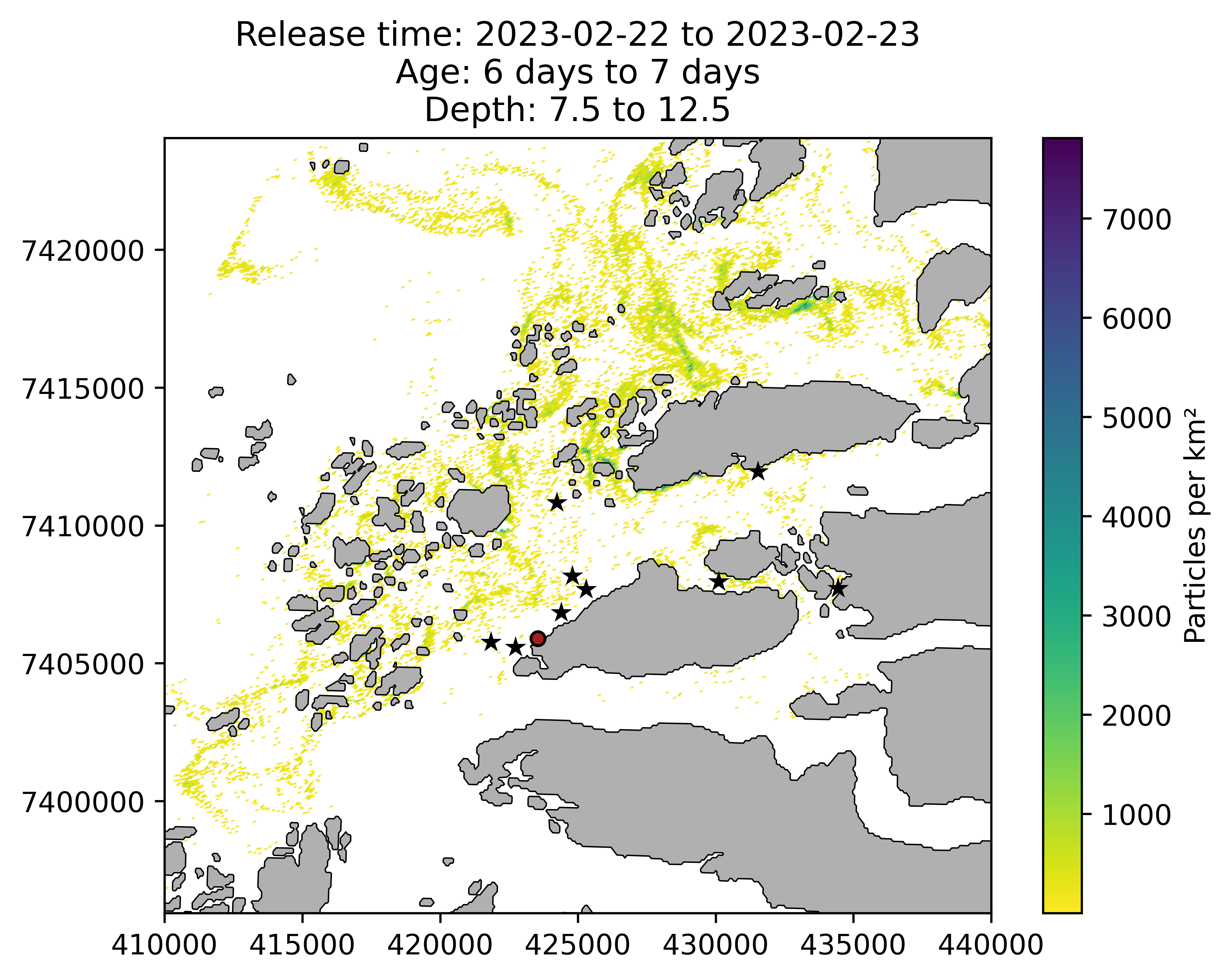


**Figure S4:** Same as figure S3, but with particles released from 10 meter depth on the previous day. Dispersion pattern is mostly northward. Note: the majority of particles remain in the outer areas and do not migrate further into the fjords.


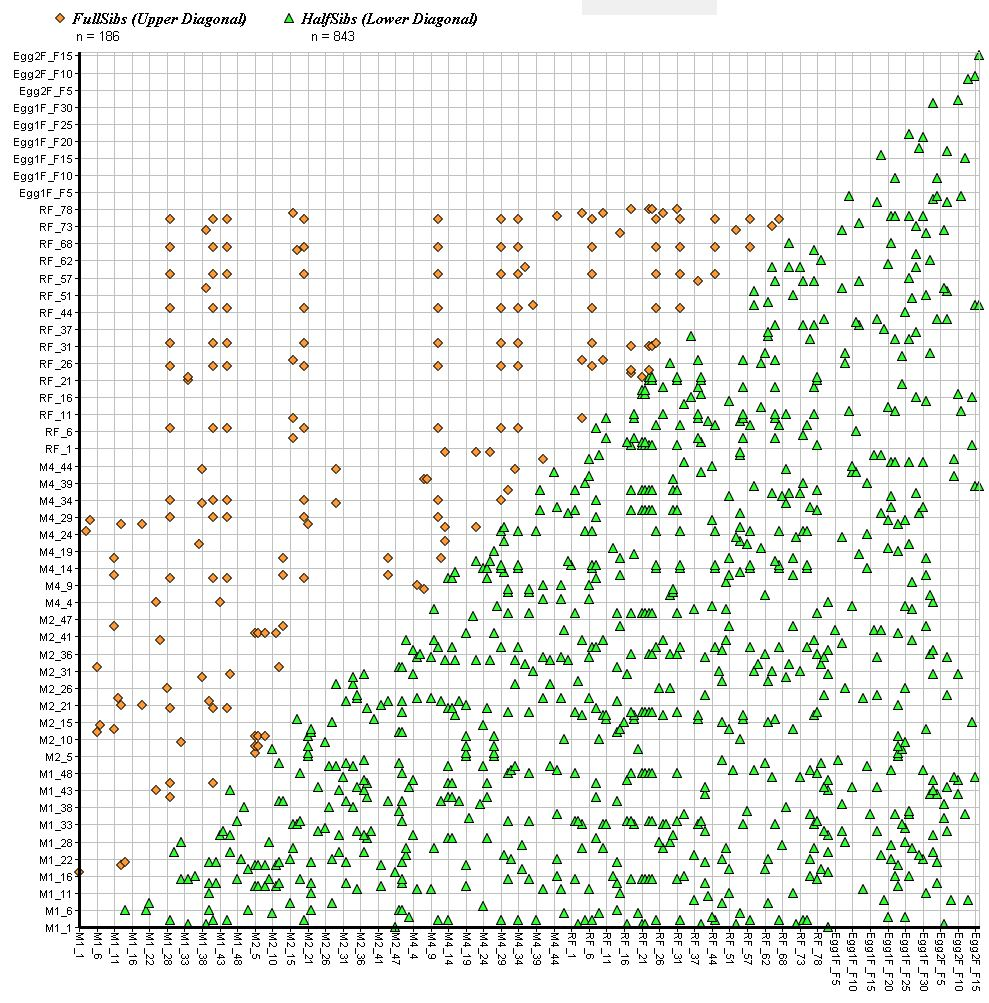


**Figure S5:** COLONY assignment plot confronting farmed fish, escapees and eggs with farmed ancestry. Orange diamonds represent estimate fullsiblings between the individuals in the corresponding coordinates whereas green triangles depict a level of relationship corresponding to halfsiblingship.

**Supplementary references**

Brooker AL, Cook D, Bentzen P, Wright JM, Doyle RW (1994) Organization of microsatellites differs between mammals and cold-water Teleost fishes. *Can J Fish Aquat Sci* **51**, 1959-1966.

Delghandi M, Wesmajervi MS, Mennen S, Nilsen F (2009) New polymorphic di-nucleotide microsatellite markers for Atlantic cod (*Gadus morhua* L.). *Cons Genet* **10**, 1037-1040.

Miller KM, Le KD, Beacham TD (2000) Development of tri- and tetranucleotide repeat microsatellite loci in Atlantic cod (*Gadus morhua*). *Mol Ecol* **9**, 238-239.

O'Reilly PT, Canino MF, Bailey KM, Bentzen P (2000) Isolation of twenty low stutter di- and tetranucleotide microsatellites for population analyses of walleye pollock and other gadoids. *J Fish Biol* **56**, 1074-1086.

Stenvik J, Wesmajervi MS, Fjalestad KT, Damsgård B, Delghandi M (2006). Development of 25 gene-associated microsatellite markers of Atlantic cod (*Gadus morhua* L.). *Mol Ecol Notes* **6:** 1105-1107

Wesmajervi MS, Tafese T, Stenvik J, Fjalestad KT, Damsgård B, Delghandi M (2007) Eight new microsatellite markers in Atlantic cod (*Gadus morhua* L.) derived from an enriched genomic library. *Mol Ecol Notes* **7**, 138-140.
